# The association of resilience with depression, anxiety, stress and physical activity during the COVID-19 pandemic

**DOI:** 10.1186/s12889-022-12911-9

**Published:** 2022-03-12

**Authors:** Quyen G. To, Corneel Vandelanotte, Kathryn Cope, Saman Khalesi, Susan L. Williams, Stephanie J. Alley, Tanya L. Thwaite, Andrew S. Fenning, Robert Stanton

**Affiliations:** 1 Appleton Institute, Central Queensland University, Rockhampton, 4701 Australia; 2 School of Health, Medical and Applied Sciences, Central Queensland University, Rockhampton, 4701 Australia

**Keywords:** Exercise, Mental health, Psychological distress, Wellbeing

## Abstract

**Background:**

COVID-19 has resulted in substantial global upheaval. Resilience is important in protecting wellbeing, however few studies have investigated changes in resilience over time, and associations between resilience with depression, anxiety, stress, and physical activity during the COVID-19 pandemic.

**Methods:**

Online surveys were conducted to collect both longitudinal and cross-sectional data at three time points during 2020. Australian adults aged 18 years and over were invited to complete the online surveys. Measures include the six-item Brief Resilience Scale, the 21-item Depression, Anxiety and Stress Scale, and the Active Australia Survey which have eight items identifying the duration and frequency of walking, and moderate and vigorous physical activities (MVPA), over the past 7 days. General linear mixed models and general linear models were used in the analysis.

**Results:**

In the longitudinal sample, adjusted differences (aDif) in resilience scores did not significantly change over time (time 2 vs. time 1 [aDif = − 0.02, 95% CI = − 0.08, 0.03], and time 3 vs. time 1 [aDif = < 0.01, 95% CI = − 0.07, 0.06]). On average, those engaging in at least 150 min of MVPA per week (aDif = 0.10, 95% CI = 0.04, 0.16), and having depression (aDif = 0.40, 95% CI = 0.33), anxiety (aDif = 0.34, 95% CI = 0.26, 0.41), and stress scores (aDif = 0.30, 95% CI = 0.23, 0.37) within the normal range had significantly higher resilience scores. The association between resilience and physical activity was independent of depression, anxiety, and stress levels. All results were similar for the cross-sectional sample.

**Conclusions:**

Resilience scores did not change significantly during the COVID-19 pandemic. However, there were significant associations between resilience with physical activity and psychological distress. This research helps inform future interventions to enhance or nurture resilience, particularly targeted at people identified as at risk of psychological distress.

## Background

Initially reported in November 2019, the novel coronavirus (COVID-19) has infected more than 244 million people worldwide, with more than 4.9 million deaths (25 October 2021) [1]. In addition to causing a global health emergency, there has been subsequent social and economic repercussions on the world’s population due to government-imposed restrictions to protect public health [2]. How people respond to a persistent stressor, such as the COVID-19 pandemic, may vary based on individual resilience levels [3], which can be defined as “the process involving an ability to withstand and cope with ongoing or repeated demands and maintain healthy functioning in different domains of life such as work and family”(p.637) [4]. Understanding resilience and how it changes across time may help in designing interventions that aim to minimise psychological distress.

Few studies have examined changes in resilience during the COVID-19 pandemic. Sturman (2020) compared levels of resilience in the United States prior to the declaration of a global pandemic (November and December 2019), to levels in the early stages of the pandemic (mid-April 2020), and found no significant change over time [5]. Similarly, Kim et al. (2020) found no significant differences in resilience of Israeli adults between the peak of the COVID-19 pandemic, when Government-enforced restrictions were imposed, and 2 months later when the restrictions had been lifted [6]. However, a USA study found that resilience in the third week of COVID-19 stay-at-home restrictions (April 2020) were lower compared to normative data collected before the pandemic [7]. Additionally, we could not identify any studies examining longitudinal changes in resilience in Australian adults.

Resilience is an important protective factor against psychological distress. A systematic review and meta-analysis found that older adults with higher resilience were less likely to have depressive symptoms [8]; however, no Australian studies were included. To our knowledge, only two studies of resilience and depression have been undertaken in Australia. One study among Iranian immigrants living in Australia found lower levels of resilience associated with higher levels of depression [9]. In contrast, another study found no significant associations between resilience and depression among homeless people in regional Australia [10]. The association between resilience and anxiety was also investigated in another study that found higher levels of resilience was associated with less anxiety among Australians aged 55–90 years [11]. Additionally, several pilot interventions that aimed to improve participants’ resilience through education workshops appeared to have positive effects in mitigating workplace stress among Australian nurses [12–14]. However, these previous studies are limited by their cross-sectional design or were not conducted among the general population. A more recent study of Australian parents reported loneliness as a significant contributor to stress during the pandemic, however high levels of social support were associated with both lower stress and anxiety [15]. Healthcare professionals are also predisposed to significant distress and anxiety, despite high levels of resilience, suggesting resilience alone is insufficient as a protective toll against poor mental health [16].

Physical activity is an important health behaviour that benefits both physical and mental health [17]. Studies conducted during the COVID-19 pandemic consistently show strong positive associations between resilience and physical activity levels [7, 18, 19]. One possible underlying mechanism for this association could be due to the positive effect of physical activity on mental health [20]. However, whether physical activity is associated with resilience, independent of mental health status, has not been investigated in these cross-sectional studies. Furthermore, to our knowledge, no Australian study of these relationships has been found.

Therefore, this study aims to investigate: 1) changes in the resilience level of Australian adults over time during the pandemic; and 2) associations between resilience with depression, anxiety, stress, and physical activity. Findings from this study contribute important insights into the role of resilience for physical activity behaviour and psychological distress among Australian adults during the pandemic.

## Methods

### Study design and participants

Online surveys were conducted to collect both longitudinal and cross-sectional data at three time points. The first survey was conducted early on during the COVID-19 pandemic from 9th to 19th April 2020; the second from 30th July to 16th August 2020; and the third between 1st and 25th December 2020. During the first time point, Australian state governments had adopted extraordinary measures to reduce the rates of infection including social distancing, lockdowns, and travel restrictions. During the second time point, all Australian states except Victoria had relaxed restrictions due to low case numbers of the infection. At the time of the third survey, most COVID-19 restrictions were lifted in all States and Territories as the rates of infection were largely under control [21, 22].

At each survey, participants (including new participants recruited for survey 2) were asked if they would like to participate in future data collection opportunities. Those completing at least two surveys became part of a longitudinal cohort while those who elected to complete only one survey formed the cross-sectional cohort. The surveys were anonymous and hosted on the Qualtrics survey platform. Australian adults aged 18 years and over were invited to complete the surveys using paid Facebook advertising, social media (e.g., Twitter) and institutional sources including email lists. Online informed consent was provided by all participants after they had read the information sheet that outlined the nature of their participation, the risks and benefits of participation, and how the data would be used. Ethical approval was granted by Central Queensland University’s Human Research Ethics Committee (Approval number 22332).

### Measures

Demographic characteristics included age (years), gender, years of schooling, weekly household income (< 1000 AUD, 1000 - < 2000 AUD, or ≥ 2000 AUD), and marital status (in a relationship or not). Chronic disease status (Yes/No) was identified using the question; “Have you ever been told by a doctor that you have any chronic health problems?”. These included one or a combination of heart disease, high blood pressure, stroke, cancer, depressive disorder, anxiety disorder, psychotic illness, bipolar disorder, diabetes, arthritis, chronic back/neck pain, asthma, COPD, and chronic kidney/renal diseases [23].

Resilience was assessed using the six-item Brief Resilience Scale (BRS). The BRS measures an individuals’ ability to bounce back from an adverse event and focuses on the ability to recover [24]. The BRS is a reliable measure of resilience, with Cronbach’s alpha ranging from 0.80 to 0.91 and a 1 month test-retest reliability (ICC) of 0.69 [24]. The BRS is comprised of six items with three positively worded items (1, 3, and 5) and negatively worded items (2, 4, and 6). For example, a positive item states “I tend to bounce back quickly after hard times” while a negative item states “I have a hard time making it through stressful events”. Responses were provided on a 5-point Likert scale with anchors at 1 (strongly disagree) and 5 (strongly agree). The scale was scored by reverse coding the negative items and then averaging the total score for the six items. Final scores range from 1.0–5.0 with a score of 3.0–4.3 considered a normal level of resilience [25].

Psychological distress was measured using the 21-item Depression, Anxiety and Stress Scale (DASS-21) [26]. The DASS-21 has shown acceptable construct validity and high reliability (Cronbach’s alphas were 0.88, 0.82 and 0.90 for depression, anxiety and stress respectively) in a non-clinical adult population [27]. Each domain has seven items scored on a 4-point Likert scale between 0 (did not apply to me at all) and 3 (applied to me very much, or most of the time). Example items were “I was aware of dryness of my mouth” or “I found myself getting agitated”. A score was calculated for each domain by adding the scores for the relevant items and multiplying by two. Standard cut-points were used to determine whether participants had symptom severity above normal for depression (≥10 points), anxiety (≥8 points), and stress (≥15 points) [26].

Physical activity was assessed using the Active Australia Survey (AAS), which comprises eight items identifying the duration and frequency of walking, and moderate and vigorous (MVPA) physical activities, over the past 7 days. For example, questions about walking are “In the last week, how many times have you walked continuously, for at least 10 minutes, for recreation, exercise or to get to or from places?” and “What do you estimate was the total time that you spent walking in this way in the last week?”. The AAS guidelines were used to calculate total physical activity by summing minutes of walking, minutes of moderate activity, and minutes of vigorous activity (multiplied by 2). Participants were then categorised as meeting the physical activity guidelines (≥150 min of moderate – vigorous (MVPA) per week) or not (< 150 min MVPA per week) [28]. The AAS criterion validity has been found to be acceptable for use in self-administered format, with correlations between self-reported physical activity and weekly pedometer steps, and accelerometry being 0.43 and 0.52 respectively [29].

### Analyses

Statistical analysis was undertaken using SAS software v9.4. Two datasets, longitudinal and repeated cross-sectional, were analysed separately. Participants completing at least two surveys were included in the longitudinal dataset. The repeated cross-sectional dataset excluded those in the longitudinal dataset and therefore included only those completing one survey. Descriptive statistics (mean, standard deviation, and percentages) were calculated and are presented for each time point. Changes in resilience scores were examined using general linear mixed models for the longitudinal data, and general linear models for cross-sectional data. In addition to bivariate analyses, estimated changes in resilience scores were also adjusted for age, gender, years of education, weekly household income, relationship status, and chronic disease status. Multiple comparison correction was applied using the simulation option in PROC GLIMMIX.

Associations between resilience scores with physical activity and depression, anxiety, and stress were also examined using general linear mixed models for the longitudinal data and general linear models for the cross-sectional data. Three models were run for both datasets. Model 1 included resilience scores, time and either physical activity, depression, anxiety, or stress. Model 2 included the additional covariates: age, gender, years of education, weekly household income, relationship status, and chronic disease status. To examine whether the observed associations were independent, physical activity, depression, anxiety, and stress were also included in Model 3 together with time and all other covariates.

Due to missing values for the household income variable being higher than 10%, analyses were conducted with and without household income as a covariate. As the results between these two analyses did not change the findings, only models including household income are presented. Crude and adjusted differences in resilience scores with 95% confidence intervals are reported. All *p*-values were two sided and considered significant if < 0.05.

## Results

Table 1 shows characteristics of the longitudinal sample. At baseline, the majority of respondents were women (68.7%) and in a relationship (64.6%), with almost half reporting a chronic disease (47.5%). On average, participants were 52.5 (SD = 14.3) years old and had about 16 (SD = 4.7) years of education. Most had scores within the normal range for depression (64.0%), anxiety (80.7%), and stress (72.9%). More than half met the physical activity guidelines (56.4%). Average resilience score was about 3.4 out of 5.0 and within the normal range (3.0–4.3). The characteristics of those in the cross-sectional sample were very similar (Table 2).

**Table 1:** Characteristics of the longitudinal sample

	Survey 1		Survey 2		Survey 3	
	n	% or mean (SD)	n	% or mean (SD)	n	% or mean (SD)
Gender						
Male	199	31.3	269	32.0	161	29.7
Female	436	68.7	573	68.1	382	70.4
Age (years)	638	52.5 (14.3)	843	53.2 (14.1)	545	53.8 (13.9)
Years of Education	638	16.5 (4.7)	843	16.6 (4.7)	545	16.5 (4.6)
Household income/week						
< 1000AUD	148	26.5	212	28.8	141	29.9
1000 – <2000AUD	176	31.5	216	29.4	135	28.6
≥ 2000AUD	234	41.9	307	41.8	196	41.5
Marital status						
Not in a relationship	223	35.4	299	35.9	203	38.0
In a relationship	407	64.6	533	64.1	331	62.0
Chronic disease						
No	335	52.5	426	50.5	264	48.4
Yes	303	47.5	417	49.5	281	51.6
Depression level						
Normal	408	64.0	536	63.6	365	67.0
Above normal	230	36.0	307	36.4	180	33.0
Anxiety level						
Normal	515	80.7	648	76.9	432	79.3
Above normal	123	19.3	195	23.1	113	20.7
Stress level						
Normal	465	72.9	640	75.9	429	78.7
Above normal	173	27.1	203	24.1	116	21.3
Meeting PA guideline						
Yes	360	56.4	460	55.0	316	58.2
No	278	43.6	377	45.0	227	41.8
Resilience score	638	3.4 (0.8)	843	3.4 (0.8)	545	3.4 (0.8)

**Table 2:** Characteristics of the cross-sectional sample

	Survey 1		Survey 2		Survey 3	
	n	% or mean (SD)	n	% or mean (SD)	n	% or mean (SD)
Gender						
Male	308	33.6	214	37.8	159	46.8
Female	609	66.4	352	62.2	181	53.2
Age (years)	925	49.4 (15.3)	571	54.2 (15.1)	348	55.6 (14.6)
Years of Education	926	16 (5.3)	573	15.8 (5.3)	349	14.9 (5.3)
Household income/week						
< 1000AUD	210	26.7	173	36.6	102	37.1
1000 – <2000AUD	225	28.6	129	27.3	78	28.4
≥ 2000AUD	352	44.7	171	36.2	95	34.6
Marital status						
Not in a relationship	356	39.4	224	40.2	140	42.9
In a relationship	547	60.6	333	59.8	186	57.1
Chronic disease						
No	498	53.8	289	50.4	198	56.7
Yes	428	46.2	284	49.6	151	43.3
Depression level						
Normal	554	59.8	312	54.5	222	63.6
Above normal	372	40.2	261	45.6	127	36.4
Anxiety level						
Normal	711	76.8	419	73.1	275	78.8
Above normal	215	23.2	154	26.9	74	21.2
Stress level						
Normal	660	71.3	411	71.7	273	78.2
Above normal	266	28.7	162	28.3	76	21.8
Meeting PA guideline						
Yes	490	54.9	291	52.7	168	50.2
No	402	45.1	261	47.3	167	49.9
Resilience score	926	3.4 (0.8)	573	3.4 (0.8)	349	3.5 (0.8)

Table 3 shows changes in resilience scores over time. In the longitudinal sample, crude (Model 1) and adjusted differences (aDif) (Model 2) in resilience scores were not significant between time 2 vs. time 1 (aDif = − 0.02, 95% CI = − 0.08, 0.03), and time 3 vs. time 1 (aDif = < 0.01, 95% CI = − 0.07, 0.06). Similarly, in the cross-sectional sample, crude and adjusted differences in resilience scores were not significant between time 2 vs. time 1 (aDif = − 0.04, 95% CI = − 0.14, 0.07), and time 3 vs. time 1 (aDif = − 0.02, 95% CI = − 0.15, 0.11).

**Table 3:** Changes in resilience scores over time (95% Confidence Interval)

	Model 1	Model 2^a^
Longitudinal		
Time 2 vs. Time 1	−0.01 (−0.07, 0.04)	−0.02 (−0.08, 0.03)
Time 3 vs. Time 1	0.01 (−0.05, 0.07)	< 0.01 (−0.07, 0.06)
Cross-sectional		
Time 2 vs. Time 1	−0.02 (−0.12, 0.07)	−0.04 (−0.14, 0.07)
Time 3 vs. Time 1	0.08 (−0.04, 0.19)	−0.02 (−0.15, 0.11)

^a^Adjusted for age, gender, years of education, household income, marital status, and chronic disease status

Table 4 shows associations between resilience scores with physical activity, depression, anxiety, and stress. On average, those engaging in at least 150 min of MVPA per week had a significantly higher resilience score (Model 2) in the longitudinal (aDif = 0.10, 95% CI = 0.04, 0.16) and cross-sectional samples (aDif = 0.19, 95% CI = 0.11, 0.27). Resilience scores were also significantly higher for those with depression scores in the normal range (longitudinal sample: aDif = 0.40, 95% CI = 0.33, 0.46; cross-sectional sample: aDif = 0.72, 95% CI = 0.64, 0.79), anxiety scores in the normal range (longitudinal sample: aDif = 0.34, 95% CI = 0.26, 0.41; cross-sectional sample: aDif = 0.68, 95% CI = 0.60, 0.77), and stress scores in the normal range (longitudinal sample: aDif = 0.30, 95% CI = 0.23, 0.37; cross-sectional sample: aDif = 0.71, 95% CI = 0.63, 0.80). Additionally, model 3 shows significant associations between resilience with physical activity, depression, anxiety, and stress, independently from one another. Specifically, resilience scores were, on average, higher for those engaging in at least 150 min MVPA per week (longitudinal sample: aDif = 0.07, 95% CI = 0.01, 0.13; cross-sectional sample: aDif = 0.15, 95% CI = 0.08, 0.21), having depression scores in the normal range (longitudinal sample: aDif = 0.30, 95% CI = 0.22, 0.37; cross-sectional sample: aDif = 0.45, 95% CI = 0.37, 0.53), anxiety scores in the normal range (longitudinal sample: aDif = 0.19, 95% CI = 0.11, 0.27; cross-sectional sample: aDif = 0.20, 95% CI = 0.10, 0.30), and stress scores in the normal range (longitudinal sample: aDif = 0.12, 95% CI = 0.04, 0.19; cross-sectional sample: aDif = 0.30, 95% CI = 0.20, 0.40).

**Table 4:** Differences (95% Confidence Interval) in resilience scores between physical activity and mental health outcomes

	Model 1	Model 2^a^	Model 3^b^
Longitudinal			
Physical activity (meeting vs. not meeting the guidelines)	0.10^***^ (0.05, 0.16)	0.10^**^ (0.04, 0.16)	0.07^*^ (0.01, 0.13)
Depression (Normal vs. above normal)	0.45^***^ (0.39, 0.51)	0.40^***^ (0.33, 0.46)	0.30^***^ (0.22, 0.37)
Anxiety (Normal vs. above normal)	0.36^***^ (0.29, 0.43)	0.34^***^ (0.26, 0.41)	0.19^***^ (0.11, 0.27)
Stress (Normal vs. above normal)	0.35^***^ (0.29, 0.42)	0.30^***^ (0.23, 0.37)	0.12^**^ (0.04, 0.19)
Cross-sectional			
Physical activity (meeting vs. not meeting the guidelines)	0.26^***^ (0.18, 0.33)	0.19^***^ (0.11, 0.27)	0.15^***^ (0.08, 0.21)
Depression (Normal vs. above normal)	0.76^***^ (0.69, 0.82)	0.72^***^ (0.64, 0.79)	0.45^***^ (0.37, 0.53)
Anxiety (Normal vs. above normal)	0.74^***^ (0.67, 0.82)	0.68^***^ (0.60, 0.77)	0.20^***^ (0.10, 0.30)
Stress (Normal vs. above normal)	0.77^***^ (0.69, 0.84)	0.71^***^ (0.63, 0.80)	0.30^***^ (0.20, 0.40)

^a^ Adjusted for time, age, gender, years of education, household income, marital status, and chronic disease status

^b^ Included physical activity, depression, anxiety, and stress together with the same covariates used in Model 2

^*^p < 0.05, ^**^p < 0.01, ^***^p < 0.001

## Discussion

This study aimed to investigate changes in resilience of Australian adults across three time points in 2020 during the COVID-19 pandemic, and the associations between resilience and physical activity, depression, anxiety, and stress. The findings show that resilience scores did not change significantly during the pandemic and that participants who engaged in at least 150 MVPA minutes per week, and with depression, anxiety, and stress scores within the normal range, had higher resilience scores. The findings were consistent between the longitudinal and cross-sectional datasets; however, the effects were larger in the cross-sectional data.

Given the extraordinary social circumstances brought about by Australian state governments to enforce movement restrictions in response to the COVID-19 pandemic, and the uncertainty as a result of the health and economic impact of the pandemic, resilience levels may have changed. However, the results from this study suggest that resilience levels largely remained stable during the pandemic, which is consistent with the results from a study in Israel [6]. This is likely due to the samples (both longitudinal and cross-sectional) including mostly Australian adults (about three quarters) with high or normal levels of resilience. Therefore, they may manage and adapt well to the impacts caused by the pandemic. Another factor may be that the Australian government was effective in responding to the pandemic (ranking 3rd among OECD countries) and providing Australians with financial support and mental health consultation via telehealth [30], and therefore helping to alleviate the impacts. It is less likely, but also possible, that levels of resilience may have decreased between pre-COVID-19 and our first survey. Unfortunately, pre-COVID-19 data are not available for comparison. However, one study comparing two cross-sectional samples in small towns in upstate New York found no significant difference in resilience between pre-COVID-19 (November and December 2019) and peak-COVID-19 (mid-April 2020) [5].

Our findings are consistent with previous studies that have found inverse associations between levels of resilience and psychological distress among patients with chronic diseases [31–33], and medical students [34, 35]. This finding is also consistent with those from other studies conducted during the COVID-19 pandemic in the U.S [36, 37] and Italy [38]. These associations were expected, as resilience reflects an individual’s ability to cope with life’s adversity, trauma, and threats; and therefore, plays a role as an adaptive defence system against psychological distress such as depression, anxiety, and stress [39]. Given their significant effects on resilience, depression, anxiety, and stress are important factors that should be considered in interventions to improve resilience level in adult populations.

Resilience was also found to be positively associated with physical activity levels in studies conducted during the COVID-19 pandemic, which is consistent with findings in the present study [7, 18, 19]. The positive effects of physical activity on resilience may occur through improving mental health and possible underlying mechanisms for this were discussed by Silverman et al. (2014) [20]. For example, physical activity could serve as a buffer against stress and stress-related disorders. Physical activity also has benefits on brain and hormonal stress-responsive systems that could improve mood and cognition [20, 40]. In this study, we found that physical activity was associated with resilience, independent of depression, anxiety, and stress levels. Although the effect size of physical activity (adjusted difference of 0.07 points) was small compared to that of depression (0.30 points), anxiety (0.19 points), and stress (0.12 points). Given that physical activity has other benefits on both physical and mental health [17], it is still an important factor for consideration in interventions targeting resilience levels.

There are a number of strengths in this study. First, the sample size is large with participants from all states and territories in Australia. Second, to the best of our knowledge, this is the first longitudinal study to explore levels of resilience during the COVID-19 pandemic in Australia. However, the study has limitations. Participation in this study was voluntary with nearly half of the sample having at least one chronic health condition and therefore, the findings may not be generalisable to populations with different characteristics. The self-reported questionnaires are also subject to recall bias, despite being validated instruments. In addition, the first survey started when the pandemic had already begun; and no pre-COVID-19 data was available. Therefore, it is not possible to know whether (and how) resilience scores changed between the pre-COVID-19 period and the first survey.

For the future, the findings from this study helps inform interventions that aim to enhance or nurture resilience. In particular, health promotion strategies that screen for, then target people identified as being at risk of psychological distress, those with low levels of resilience, or those not meeting the physical activity guidelines may maximize the effects of the interventions. Primary health care providers, Government websites, not-for-profit, or other mental health services could provide rapid screening then direct people to appropriate care.

## Conclusions

Resilience scores did not change significantly during the COVID-19 pandemic. Participants who met the physical activity guidelines, had depression, anxiety, and stress scores within the normal range, had higher resilience scores compared to those who were less active and those with more psychological distress. Maintaining healthy behaviours such as regular physical activity may buffer the adverse psychological effect of the pandemic and maintain mental health and wellbeing.

## Data Availability

The datasets used and/or analysed during the current study are available from the corresponding author on reasonable request.

## Abbreviations

AAS: the Active Australia Survey
BRS: The Brief Resilience Scale
DASS-21: the Depression, Anxiety, and Stress Scale
MVPA: Moderate and Vigorous Physical Activity
OECD: Organisation for Economic Cooperation and Development
